# Gene therapy in cardiac and vascular diseases: a review of approaches to treat genetic and common cardiovascular diseases with novel gene-based therapeutics

**DOI:** 10.1093/cvr/cvaf109

**Published:** 2025-08-25

**Authors:** Patricia L Musolino, Susan J Rosser, Mairi Brittan, David E Newby, Colin Berry, Paul R Riley, Mauro Giacca, Roger J Hajjar, Andrew H Baker

**Affiliations:** Center for Genomic Medicine, Massachusetts General Hospital, Harvard Medical School, 55 Fruit St. Lunder Building 6th Floor Boston, MA 02114; UK Centre for Mammalian Synthetic Biology at the Institute of Quantitative Biology, Biochemistry, and Biotechnology, School of Biological Sciences, University of Edinburgh, Edinburgh, UK; British Heart Foundation Centre for Research Excellence, Centre for Cardiovascular Science, Queens Medical Research Institute, 47 Little France Crescent, University of Edinburgh, Edinburgh EH16 4TJ, UK; MRC/BHF Centre of Research Excellence in Advanced Cardiac Therapies (REACT), 7 Little France Crescent, University of Edinburgh, Edinburgh EH16 4TJ, UK; British Heart Foundation Centre for Research Excellence, Centre for Cardiovascular Science, Queens Medical Research Institute, 47 Little France Crescent, University of Edinburgh, Edinburgh EH16 4TJ, UK; MRC/BHF Centre of Research Excellence in Advanced Cardiac Therapies (REACT), 7 Little France Crescent, University of Edinburgh, Edinburgh EH16 4TJ, UK; BHF Glasgow Cardiovascular Research Centre, University of Glasgow and Golden Jubilee National Hospital, Clydebank, UK; Institute of Developmental & Regenerative Medicine (IDRM), IMS-Tetsuya Nakamura Building, Old Road Campus, University of Oxford, Oxford OX3 7TY, UK; MRC/BHF Centre of Research Excellence in Advanced Cardiac Therapies (REACT), IMS-Tetsuya Nakamura Building, Old Road Campus, University of Oxford, Oxford OX3 7TY, UK; School of Cardiovascular and Metabolic Medicine & Sciences and British Heart Foundation Centre of Research Excellence, King’s College London, James Black Centre125 Coldharbour Lane, London SE5 9NU, UK; MRC/BHF Centre of Research Excellence in Advanced Cardiac Therapies (REACT), King’s College London, James Black Centre125 Coldharbour Lane, London, SE5 9NUL; Gene & Cell Therapy Institute, Massachusetts General Brigham, Cambridge, MA 02139, USA; British Heart Foundation Centre for Research Excellence, Centre for Cardiovascular Science, Queens Medical Research Institute, 47 Little France Crescent, University of Edinburgh, Edinburgh EH16 4TJ, UK; MRC/BHF Centre of Research Excellence in Advanced Cardiac Therapies (REACT), 7 Little France Crescent, University of Edinburgh, Edinburgh EH16 4TJ, UK; Department of Pathology and CARIM School for Cardiovascular Diseases, Maastricht University, UNS 50, Universiteitssingel 50, Maastricht 6229ER, The Netherlands

**Keywords:** Gene therapy, Cardiovascular disease, Heart failure, Vascular, Gene therapy vectors, Regeneration, Gene editing

## Abstract

In the past decade, there has been substantive progress in gene therapy across disease indications. However, despite multiple gene therapies being approved for clinical use, none have a cardiovascular indication. Several reasons for this have inhibited or delayed progress in the cardiovascular field. First, developing cardiovascular gene therapeutics represents a substantial technical challenge, particularly relating to identifying and building effective delivery systems for therapeutic cargo that will be sufficient to gain meaningful efficacy with acceptable safety for the patient. Second, for genetic disease, gene editing therapy of pathogenic variants is at a relatively early stage of development. Third, since this is a field in development, the optimal design of clinical trials of cardiovascular gene therapies is also evolving and requires expert attention. Despite this, recent and current clinical trials are charting new ground, gaining valuable new patient-focused information that provides critical new learning and bench-to-bedside iterative development that has been so successful in other disease areas. While most clinical trials currently focus on cardiac gene therapy, vascular approaches are being developed, both genetic and common. We herein review the state-of-the-art in this rapidly progressing field of study. We consider gene therapy vector design, including transcriptional control, an area of incredible opportunity through engineering biology approaches to design, build, and test bespoke transcriptional units for expression of therapeutic cargo. Achieving progress in this exciting field will require close working between all stakeholders, including academic, clinical, industry, regulatory, and patient communities. Based on current progress, there is a 10-year horizon for bringing several cardiovascular gene therapies to licensing.

## Graphical Abstract

**Graphical Abstract:**
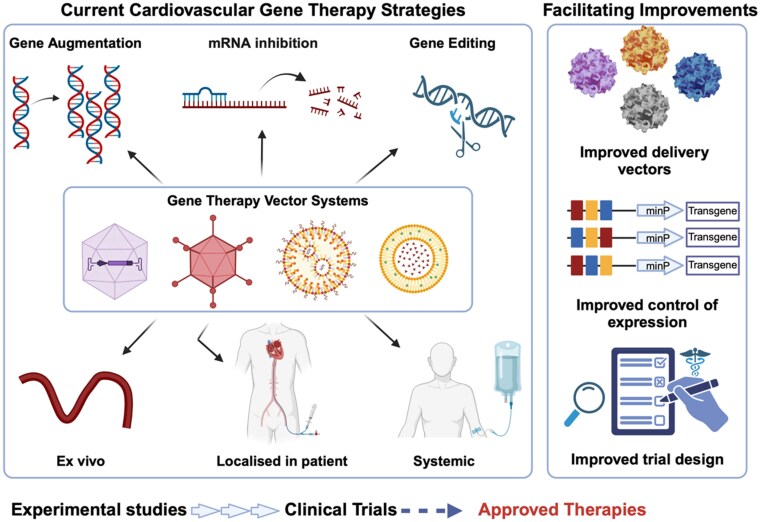
Clinical applications of gene therapy in the cardiovascular system. The opportunities range from genetic editing for cardiomyopathies to new approaches for regenerating the heart and treatment of vascular remodelling via gene augmentation or inhibition. Each application requires a bespoke approach to achieve appropriate clinical benefits for patients through delivery of therapeutic cargo and control overexpression of genetic medicines.

## 1. Introduction

This review focuses on gene therapies that are being developed for cardiovascular disease. Gene therapy is an advanced therapy medicinal product. Other advanced therapy approaches include cell-based patches, associated biomaterials, and RNA therapies that are being applied in the cardiovascular setting and are covered in excellent recent reviews.^1–4^

In this article, we present a clinical perspective on gene therapy for cardiovascular disease. We describe the gaps between preclinical experimental studies and clinical gene therapy for cardiovascular disease and highlight the solutions that are being developed. We additionally provide a brief historical perspective and summarize of the technologies currently available for gene delivery. With respect to diseases, heart failure is considered first and then vascular disease. Considering heart failure, the specific topics are (i) targeting pathways to treat heart failure, (ii) treating genetic cardiomyopathies, and (iii) targeting cardiac regeneration. Considering vascular disease, we discuss the opportunity for *ex vivo* vascular gene therapy, then gene editing technologies for the heart and vasculature. Approaches that are being developed to improve control of expression of the therapeutic transgene/cargo in gene therapies once in the cell are also discussed.

Gene therapy medicines are described by the European Medicines Agency as follows: ‘*These [medicines] contain genes that lead to a therapeutic, prophylactic or diagnostic effect. They work by inserting “recombinant” genes into the body, usually to treat a variety of diseases, including genetic disorders, cancer or long-term diseases. A recombinant gene is a stretch of DNA that is created in the laboratory, bringing together DNA from different sources*’. Historically, the first cardiovascular gene therapy publication was in 1990, and this demonstrated the feasibility of gene delivery to the blood vessel,^5^ catalysing the field to pursue new approaches in monogenic disease and other complex cardiovascular diseases. There has been an intense effort to evolve efficient delivery systems that adequately allow gene manipulation for efficacy gain using advanced therapies. These include RNA therapies such as antisense oligonucleotides, N-acetylgalactosamine ligand-modified systems, short interfering RNA, and microRNA modification using lipid nanoparticles and viral vector systems including adenovirus- and adeno-associated virus (AAV)–based vectors. In the cardiovascular setting, the main modalities for gene therapy delivery are based on viral vectors, particularly recombinant adenovirus-based vectors, those based on adenovirus, AAV, and non-viral systems such as lipid nanoparticles. Attributes and limitations of each approach are relevant. For example, AAV vectors have high affinity for muscle, including cardiac muscle, yet these vectors have limited capacity for cloning genes (approximately 4.7 kb of DNA). Adenovirus-based vectors have attributes including the ability to transfer genetic material to a broader range of cell types and a larger cloning capacity (up to 36 kb), yet can be more immunogenic. The historical development of these technologies is covered in other reviews.^6–9^ In this article, we describe how gene delivery vectors are being used currently in the cardiovascular setting and what developments are enabling improvements in gene delivery that will have an impact on clinical translation.

Like other disease areas, advances in the field from experimental-to-clinical studies were rapid but challenges remained. They included limited options and efficacy of delivery systems in cardiovascular tissue, sometimes inadequate knowledge of disease pathogenesis and limitations in the design of clinical trials. Major safety events in clinical trials of gene therapy disseminated the field in the late 1990s and early 2000s. One historical and high profile example is the tragic death of Jesse Gelsinger following adenovirus-based vector gene therapy infusion for ornithine transcarbamylase deficiency.^10^ Another key example was the induction of leukaemia following gene therapy for immunodeficiency disease.^11^ This may be explained by the uncontrolled integration of genetic material into host DNA by retroviral-based vector systems.^12^

These serious adverse events moved the field to improve the methods for gene delivery in vivo and related safety monitoring as well as improved clinical trial design. New advances emerged for monogenic diseases such as haemophilia, ocular disease, neurological conditions, and childhood immunedeficienies.^12–17^ These developments re-invigorated the field, both academically and commercially, and there are now over 20 Federal Drug Agency or European Medicines Agency approved genetic medicines. These advances led to renewed optimism for cardiovascular gene therapy.

## 2. Gene therapy to the myocardium

A major challenge for cardiac gene therapy is delivery modality. Non-selective systemic delivery of therapies for the heart is relatively simple (and used widely in experimental studies) but requires high doses since much of the delivery vector disseminates throughout the body. This systemic approach necessitates a regimen of concomitant or on-demand immunosuppression in humans to mitigate the antiviral host responses that are characteristic of AAV-mediated gene delivery regimens. The complications from high-dose delivery of AAV vectors have been evident in muscular dystrophy clinical trials where liver failure and immune reactions resulting in acute respiratory distress syndrome (ARDS) have, tragically, led to death.^18,19^ Severe but reversible adverse events (SAEs) have also been observed in cardiac gene therapy trials where systemic injection of AAV9 has been used highlighting key areas for caution on intravenous dosing for risk: benefit considerations for the patient.^20,21^ High-dose, systemic regimens of AAV9 therapy push the limits of manufacture and cost-of-goods concerns. In contrast, intracoronary AAV9 delivery requires much lower doses (∼20–100 times lower than intravenous) without the use of immunosuppressants. This can result in high expression with strong cardiac tropic vectors, such as the novel AAV called AAV2i8,^22^ which are discussed more below as this vector shows improved uptake into heart tissue. However, improvements in gene delivery vectors for more efficient cardiac gene delivery are needed.

Intracoronary delivery of gene therapy involves relatively simple cardiac catheterization, and this has been safely achieved in more than 300 patients with severe heart failure in the AAV1.SERCA2a trials.^23,24^ When considering intracoronary versus intravenous delivery, the transgene, disease state, clinical trial design, and cost of goods all need to be taken into account. The critical step for clinical therapy development is demonstrating that the approach delivers sufficient vector to the target tissue with the benefit being greater than the risk. With the future development of potentially more efficient cardiac tropic vectors, including lipid nanoparticle-based systems and new AAV vectors, systemic and intracardiac delivery at low doses of vectors should be possible to facilitate clinical gene therapy development through improved delivery of gene therapy.

Considering new developments, Ishikawa *et al*. showed that using mechanical support (such as the Impella device) can increase AAV myocardial uptake in the myocardium of swine models by 10^6^-fold compared to intracoronary delivery. Utilizing mechanical support for cardiac gene delivery offers a clinically applicable strategy.

### 2.1 Targeting heart failure

The population of patients suffering from heart failure is segmented into reduced ejection fraction (HFrEF) and preserved ejection fraction (HFpEF). For both HFrEF and HFpEF, there is an unmet clinical need for new therapies that heal or reverse cardiac damage.^26^

In HFrEF, the dominant vector for cardiac gene therapy has been AAV and the different types available, some of which have favourable virus uptake into muscle, including the myocardium (principally serotypes AAV1, AAV6, and AAV9). We discuss below the clinical data to date and the steps that are being taken to maximize this opportunity.

Several clinical trials involve interventions targeting intracellular calcium cycling abnormalities that characterize failing human cardiac myocytes.^27^ The cardiac sarcoplasmic reticulum (SR) calcium ATPase pump (SERCA2a) is deficient in terms of function and expression in heart failure associated with Duchenne muscular dystrophy (DMD) myopathy.^28^ As such, DMD has been targeted in experimental gene therapy studies which eventually led to clinical studies.^24,29–31^ Clinical trials targeting SERCA2a by delivering AAV1.SERCA2a via intracoronary infusion in patients with heart failure progressed to Phase 2b (∼250 patients), but failed to demonstrate therapeutic efficacy.^32,33^ In all these trials, AAV1.SERCA2a was delivered at a dose of 10^13^ virus genomes (vg) per patient and gene expression analysis of myocardial biopsies from study participants indicated that less than 1% of cardiomyocytes were transduced.^32–34^

Informed by this experience, there are three current clinical trials evaluating higher doses of AAV1.SERCA2a with the aim of enhancing myocardial vector uptake. The AAV doses are 3 × 10^13^ vg and 4.5 × 10^13^ vector genomes of AAV per patient in the heart failure with reduced ejection fraction (MUSIC-HFrEF), heart failure with preserved ejection fraction (MUSIC-HFpEF), and Duchenne muscular dystrophy cardiomyopathy (MUSIC-DMD) trials. Notably, in the MUSIC-HFpEF trial, patients will have measurements of pulmonary capillary wedge pressure at rest, during exercise and then in recovery and repeatedly assessed at baseline and 6 and 12 months post gene transfer.^35^ MUSIC-DMD is a Phase 1b study, and the aim is to assess the safety and potential efficacy of AAV1.SERCA2a, administered as a one-time antegrade epicardial coronary artery infusion.^35^

Calcium cycling is also a target for gene therapy. A Phase 1 clinical trial in non-ischaemic cardiomyopathy involves a novel AAV2i8 capsid that delivers a constitutively active inhibitor (I-1c) of protein phosphatase 1 (I-1) via a single intracoronary infusion of the vector. The AAV2i8 capsid was designed by combining AAV2 and AAV8 by Asokan and Samulski^22,36–38^ to improve AAV-mediated gene delivery to the heart. Gene therapy uptake via the capsid has been evidenced by infectivity ∼1 vg/cardiac cell in a human left ventricular sample 13 months post gene transfer.^22^ More evidence will be needed to clarify whether myocardial uptake by this approach is clinically beneficial. In a new development, a Phase 2 clinical trial of AAV2i8.I-1c at two doses vs. placebo (GENEPHIT) is currently under way.^22^

### 2.2 Targeting pathways to treat genetic cardiomyopathies

**Figure 1:**
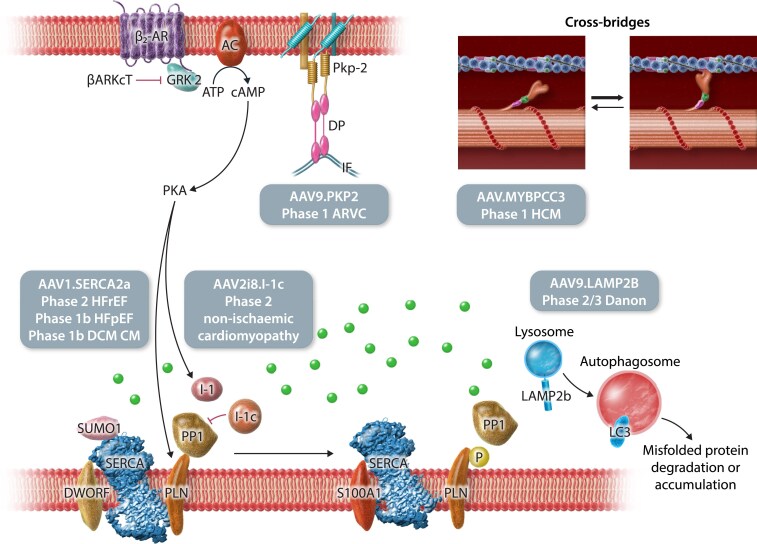
Illustration of gene therapy targets in cardiac myocytes. Different strategies to modify cardiomyocyte function converge on improving calcium handling and genetic mutations. Intracoronary delivery of AAV1.SERCA2a delivery is being tested in Phase 1/2a for the treatment HFrEF, HFpEF, and DMD cardiomyopathy and intracoronary of AAV2i8.I-1c has entered Phase 2 for the treatment of non-ischaemic cardiomyopathy. Intravenous delivery of AAV9.LAMP2b has completed enrolment in its pivotal trial in patients with Danon. Intravenous delivery of AAV9.PKP2 is in Phase 1 for ARVC while intravenous delivery of AAV.MYBPC3 for hypertrophic cardiomyopathy. SERCA2a, cardiac isoform of the sarcoplasmic reticulum calcium ATPase; SUMO1, small ubiquitin modifier 1; PLN, phospholamban; DWORF, dwarf open reading frame; S100A1, S100 calcium binding protein A1; PP1, protein phosphatase 1; I-1, inhibitor 1; I-1c, constitutively active form of I-1; GRK2, G protein-coupled receptor kinase 2; AC, adenylyl Cyclase; βARKCT, beta adrenergic receptor kinase carboxyl-terminus; MYBPC3, myosin binding protein C3; LAMP2B, lysosome associated membrane protein 2; PKP2, plakophilin-2; DP, desmoplakin; IF, intermediate filament; HFrEF, heart failure with reduced ejection fraction; HFpEF, heart failure with preserved ejection fraction; DMD, Duchenne muscular dystrophy.

Genetic cardiomyopathies include sarcomeric, syndromic, inborn error of metabolism, storage, and other forms of myocardial disease, including transthyretin (TTR) amyloidosis (refer to *Figure 1*). Gene therapy strategies aimed at blocking TTR gene expression with CRISPR/Cas9 genome editing are being evaluated in ongoing clinical trials.^39^ In a follow-up study, a single dose of this therapy appeared to be safe and was associated with consistent and clear reductions in serum TTR levels.^40^ The authors stated that this induced evidence of limited disease progression throughout treatment, but lacked pivotal data in this Phase I, open-label trial. The ongoing Phase 3 MAGNITUDE trial will likely provide key clinical data on efficacy.^41^ Even though the efficient targeting in cardiac amyloidosis has been to the liver, the effects on cardiac function have been encouraging, highlighting a novel liver–heart axis. Since liver uptake of gene therapy may be considerable, this liver–heart axis has potential for enhancing GalNAc or the liver tropic AAV-8 virus, as vehicles for cardiac effects.

Inherited cardiomyopathies are obvious candidates for traditional gene therapy approaches (i.e. gene augmentation or inhibition) or gene editing (to correct the mutation) (*Table 1*). There are many examples of early phase clinical trials in this area, with safety being critical. Danon disease (LAMP2 deficiency) is an X-linked dominant lysosomal storage disease with a phenotype of cardiac hypertrophy in adolescent males who may eventually require heart transplantation. LAMP2B is a mediator of autophagy and its deficiency results in intracellular vacuoles, myofibrillar disarray, hypertrophy, and fibrosis in cardiac tissues. A dose-escalation Phase 1 clinical trial using intravenous delivery of AAV9.LAMP2B in patients with Danon disease was recently presented.^42^ Patients were followed for 24–54 months post injection. LAMP2 protein expression was increased and associated with beneficial effects on left ventricular mass index, preservation of left ventricular ejection fraction, and cardiac biomarkers including troponin I and N-terminal pro-B-type natriuretic peptide.^42^ However, despite aggressive immunosuppression to mitigate responses to high-dose AAV, severe adverse events were observed in some of the patients treated.

**Table 1:** Hereditary cardiac indications leveraging AAV vectors

Disease	Phenotype	Overall prevalence	Causative gene	AAV	Mode of delivery	Clinical trial
Danon	X-linked recessive hypertrophic cardiomyopathy	15–30 k (US + EU)	LAMP2^a^	AAV9	Intravenous	Phase 1 completed with two high doses in adult and paediatric patients Pivotal Phase 2 started
Genetic hypertrophic cardiomyopathy	Severe hypertrophy of the walls of the ventricles	>500 k (US, total HCM)	MYBPC3^b^	AAV9	Intravenous	Phase 1 trial ongoing intravenous. High dose
Arrhythmogenic right ventricular cardiomyopathy (ARVC)	Arrhythmias and right ventricular dyskinesis. Majority due to PKP2 mutations	∼100 K in US	PKP2^c^	AAVrh10 AAVrh74	Intravenous	Dose-escalation Phase 1 trial intravenous. High dose
Friedreich’s ataxia	Autosomal Recessive neurological and muscular disorder affecting the heart	∼6000 in US	FXN^d^	AAVrh10	Intravenous	Dose-escalation Phase 1 trial intravenous. High dose
Duchenne cardiomyopathy	Autosomal dominant, LVEF < 40%	∼25 000 in US	SERCA2a^e^	AAV1	Intracoronary	Dose-escalation Phase 1 trial

^a^LAMP2: lysosomal associated membrane protein 2.

^b^MYBPC3: Myosin Binding Protein C3.

^c^PKP2: Plakophilin-2.

^d^FXN: frataxin.

^e^SERCA2a: cardiac isoform of the sarcoplasmic reticulum calcium ATPase.

Recently, the FDA approved the combined endpoints of *LAMP2B* expression + LV mass reduction for a 12-patient pivotal trial for Danon disease. This pivotal trial in cardiac gene therapy has completed enrolment and is awaiting follow-up. This clinical trial may represent a reference benchmark for future trials of trials in the field.

Second, arrhythmogenic right ventricular cardiomyopathy (ARVC) is caused by PKP2 pathogenic variants in 20–46% of cases where they are associated with increased risk of sudden cardiac death in those under 30 years of age. In experimental studies, AAV gene therapy of PKP2 has rescued models of ARVC secondary to PKP2 knockdowns or pathogenic variants. In another new development, a Phase 1 trial of AAV gene therapy of PKP2 in patients with ARVC secondary to PKP2 loss of function variants is ongoing.

Third, myosin heavy chain (MHC) variant-related cardiomyopathies are genetically associated with MYH6/7 or MYBPC3 variant. MYBPC3 pathogenic variants are the most common cause of hypertrophic cardiomyopathy (HCM), accounting for about half of identified pathogenic variants.^43–46^ A Phase 1 trial delivering MYBPC3 in HCM has been initiated.

Finally, with RBM20-associated dilated cardiomyopathies (DCMs), patients with pathogenic RBM20 pathogenic variants present at late stages of cardiac failure with debilitating symptoms along with ventricular arrhythmias. RBM20 pathogenic variants cluster within an arginine/serine-rich (RS-rich) domain, mediating nuclear localization.^47–50^ These variants induce RBM20 mis-localization to form abnormal ribonucleoprotein granules in the cytoplasm of cardiomyocytes and abnormal alternative splicing of cardiac genes, contributing to DCM. In experimental models, AAV gene therapy for gene editing of RBM20 pathogenic variants rescues cardiac dysfunction and decreases ventricular arrhythmias.^49^

Early observations of improved AAV delivery to human myocardium are encouraging, and ongoing work in progress will provide a rich learning resource in the coming years.

### 2.3 Targeting cardiac regeneration

**Figure 2:**
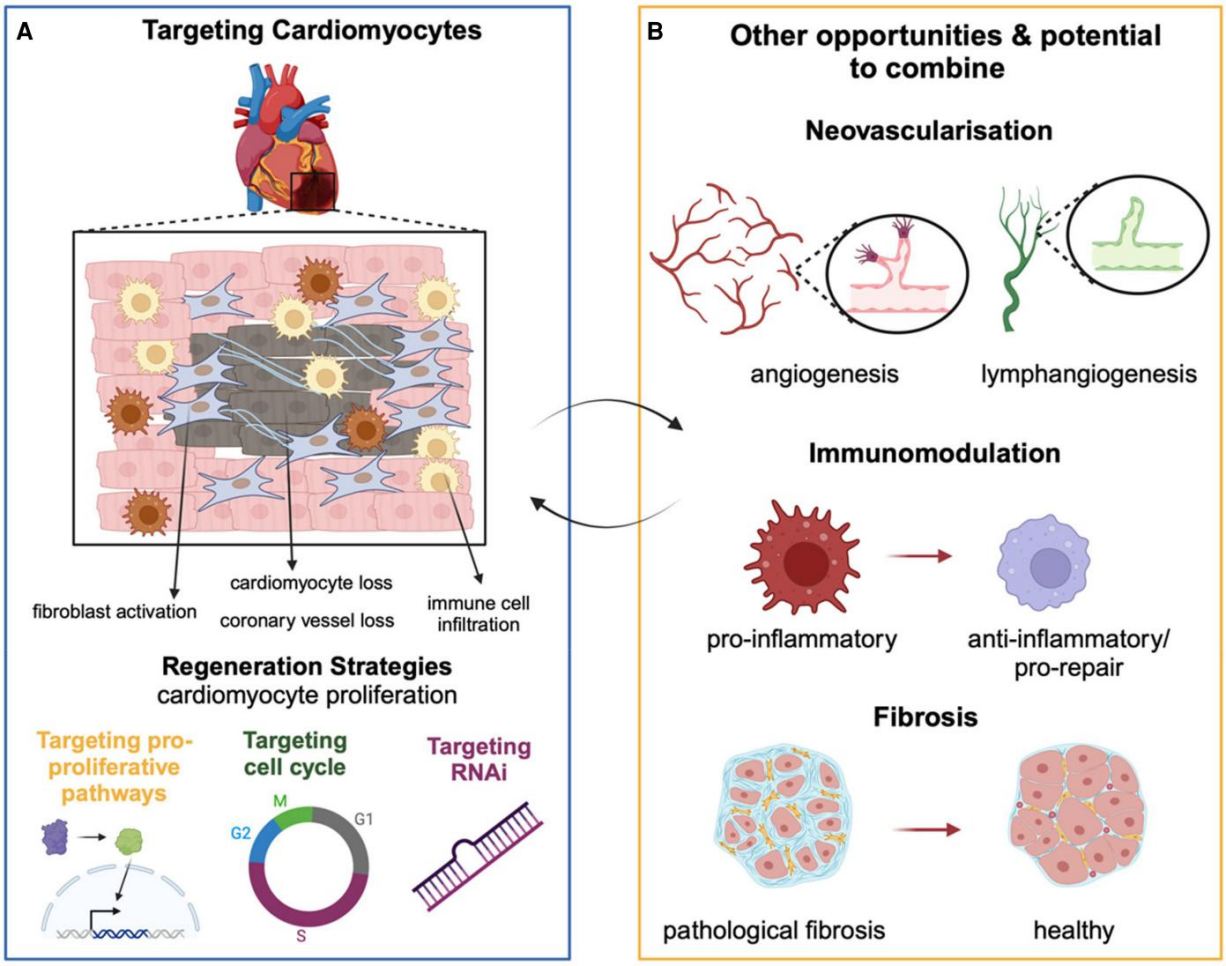
Main targets for cardiac regeneration. (*A*) Cardiac injury results in cardiomyocyte loss, reactive fibroblasts activation, and tissue infiltration with inflammatory and immune cells. Myocardial regeneration requires *de novo* formation of cardiomyocytes. This process can be stimulated by the activation of one of the pro-proliferative pathways that can be active in these cells (e.g. Hippo pathway deactivation followed by Yap activation, or activation of Notch or beta-catenin), or by direct targeting of cell cycle regulators (e.g. overexpression of cyclin A), or by exploiting the RNA interference pathway by delivering pro-proliferative miRNAs (e.g. miR-199a-3p, miR-302-3p, miR-1825 or others). (*B*) Beyond cardiomyocyte proliferation, myocardial regeneration requires and is regulated by formation of new blood and lymphatic vessels, modulation of the inflammatory response towards a regenerative state, and regulation of excessive fibrotic deposition.

Gene therapy also has potential to improve heart function by stimulating the generation of new cardiac tissue (see *Figure 2*). Cardiomyocyte loss and lack of regeneration are features of HFrEF, especially following myocardial infarction (MI).

One strategy has focused on the activation of pathways that control cardiomyocyte proliferation during development. For example, in experimental studies, overexpression of Yes1 Associated Regulatory Protein (YAP), using an AAV9 vector^51^ or shRNA knockdown of Salvador Homolog 1 (SAV1), a co-factor of the YAP inhibitory kinase MST1/2 (Hippo in *Drosophila*) in mouse hearts with HF after MI,^52^ induces cardiac regeneration.^53^

A second regenerative medicine approach is to manipulate the cell cycle with gene therapy. Strategies include overexpressing positive cell cycle regulators such as cyclin A2,^54^ the metabolic enzyme PKM2 using modified RNA,^55^ the simultaneous delivery of CDK1, CDK4, cyclin B1, and cyclin D1 using gene therapy^56^ or by indirectly targeting proliferation by administering modified RNA coding for the metabolic enzyme PKM2.^55^ Several microRNA (miRNA) genes can also stimulate cardiomyocyte proliferation (reviewed in ref. Braga *et al*.^57^). In a mouse MI model, administration of miR-199a or miR-590a,^58,59^ miR-294, a member of the miR-302 superfamily,^60^ or miR-19a/19b^61^ provided experimental evidence of cardiac regeneration and improvement of cardiac function. However, activity of these miRNAs should be controlled, as their prolonged activation using AAV vectors can lead to hyperproliferation and adverse outcomes, as observed for AAV6-miR-199a in pig models.^62^ These observations underscore the need to control the extent of gene expression in cardiovascular tissues. The concept of control of transgene transcription is discussed later.

Most clinical trials targeting improvements in cardiac vasculature, despite demonstrating safety and feasibility, have proven disappointing despite demonstrating safety and feasibility,^63^ thus highlighting the need for further innovation. One such approach is the development of an adenovirus-based approach called XC001 that is designed to increase myocardial expression of the predominant three VEGF isoforms following gene transfer. EXACT was a single-arm, multicentre, open-label clinical trial that enrolled 32 patients with refractory angina to receive transepicardial delivery of virus. The preliminary findings from this study provide some support for the safety and efficacy (e.g. improvement in angina class) of this approach.^64^ However, the clinical significance should next be assessed in a future double-blind, placebo controlled trial that has adequate power to provide reliable information on safety and efficacy.

The cardiac lymphatic system may represent a novel target for gene therapy post-MI. Lymphatics in the heart myocardial fluid balance and immune cell homeostasis and lymphatic function can be impaired post-MI.^65^ Experimental studies of gene therapy for lymphatic vessels have shown a significant and prolonged expansion of lymphatic vessels post-MI.^65^ As a possible gene therapy, AAV-mediated delivery of VEGFC-C165, which specifically targets VEGFR3, improved experimental MI outcomes.^66^ The VEGF family plays critical roles in development and maintenance of functional vascular and lymphatic networks,^67,68^ and VEGF-C is also thought to act as mitogen for adult lymphatic endothelial cells^69,70^ as well as regulating post-natal angiogenesis.^65^ As such, gene therapeutic strategies to augment both vascular and lymphatic responses post-MI merit further evaluation (*Figure 2*). A strategy that combines targeted gene therapy for fibrosis and inflammation merits experimental evaluation in the first instance (*Figure 2*).

## 3. *Ex vivo* vascular gene therapy

**Figure 3:**
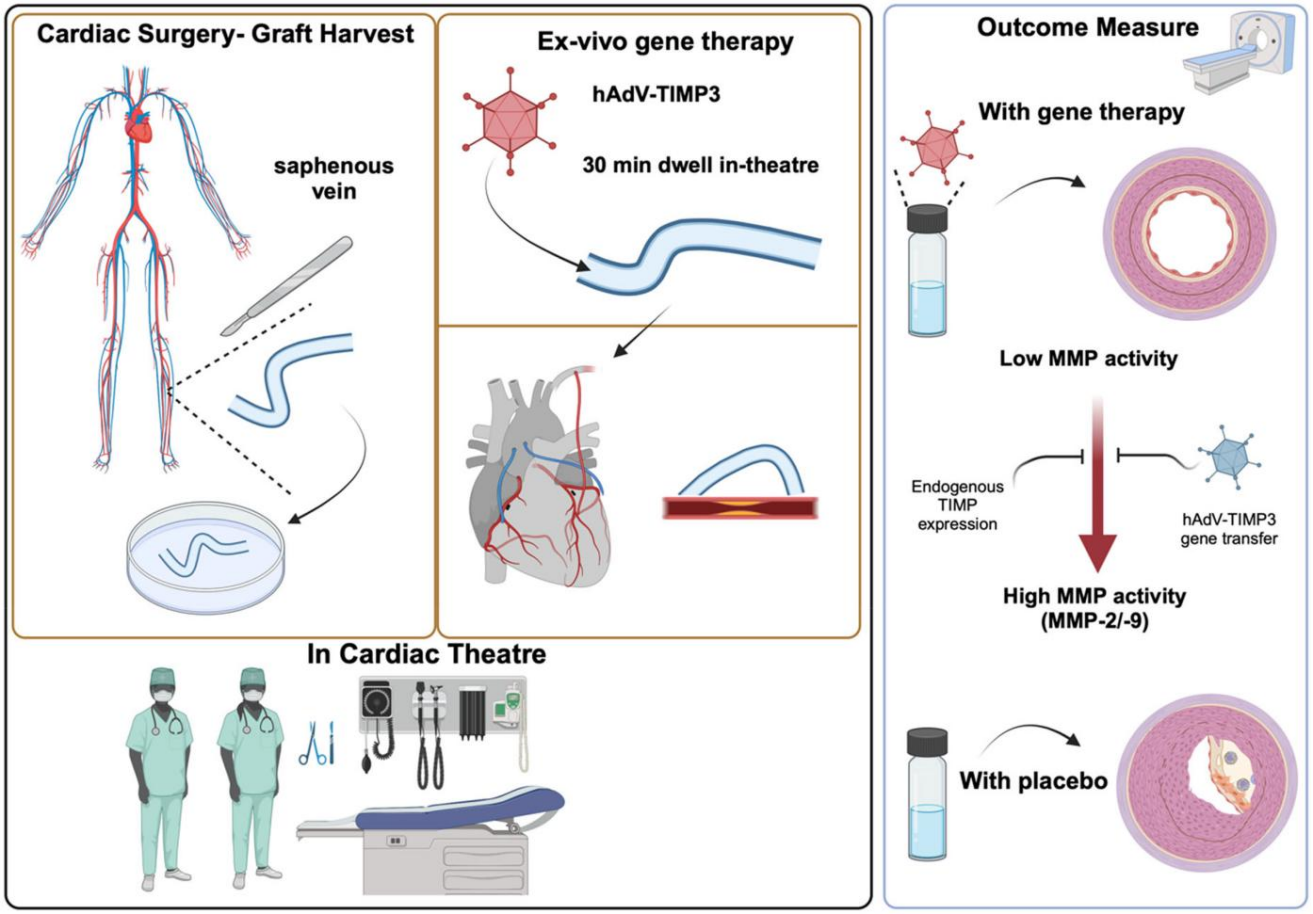
*Ex vivo* gene therapy during coronary artery bypass graft surgery to prevent late graft failure. During cardiac surgery, the harvested autologous saphenous vein is cannulated and infused with recombinant adenovirus to create a luminal dwell, in the absence of additional pressure to the vein. Gene transfer occurs in the next 30 min prior to washing the graft gently to remove excess adenovirus. Following grafting, the cells in the vein graft wall that are infected by the adenovirus will express the cloned gene, tissue inhibitor of metalloproteinase-3 (TIMP-3) that is secreted from those cells. Excess TIMP-3 acts to prevent extracellular matrix degradation by matrix metalloproteinase enzymes, leading to prevention of intimal hyperplasia. A forthcoming Phase 1 clinical trial in coronary artery bypass graft surgery (PROTECT, ISRCTN43650325) aims to assess the safety of this approach.

*Ex vivo* gene therapy is a novel approach for vein graft failure (see *Figure 3*). The surgical treatment of coronary and peripheral artery disease includes bypass conduits for coronary and lower limb peripheral artery bypass grafting applications, which largely still involve the use of autologous saphenous vein grafts. The success of surgery is limited by vein graft occlusion due to barotrauma and accelerated atherosclerosis. Thus, strategies to limit vascular damage in the early aftermath of vein grafting would, in theory, prevent long-term failure by blocking the development of vein graft atheroma. A host of mechanisms could be targeted as there is a tremendous breadth of experimental research that has identified causal mechanisms.

One such approach nearing clinic is the use of adenoviral vectors to overexpress TIMP-3 delivered in this manner, which block adverse remodelling induced my tissue injury. This has been shown in human saphenous vein tissue *ex vivo* and in pig grafts *in vivo* in experimental studies.^71,72^ A clear advantage of this approach is limiting the patient to exposure to the gene therapy vector system since the gene delivery occurs *ex vivo* and the excess vector can be washed from the graft before grafting. A first-in-human, placebo-controlled, dose-ranging clinical trial of Ad5TIMP3 gene therapy given *ex vivo* to saphenous vein immediately prior to aorto-coronary bypass grafting, PROTECT, is due to begin in the Golden Jubilee National Hospital (Glasgow, UK), to assess the safety and feasibility of this approach (clinical trial registration: ISRCTN43650325; https://www.isrctn.com/ISRCTN43650325) (*Figure 3*).

## 4. Gene editing technologies for the heart and vasculature

Genome editing technologies enable the permanent modification of DNA sequence in living cells and thus have the capacity to correct DNA mutations that cause cardiovascular disease. This process has been simplified by the discovery that CRISPR-Cas nucleases such as Cas9 can be readily programmed to edit specific DNA sites using a guide RNA (gRNA).^73,74^ In complex with the gRNA, the Cas9-gRNA ribonucleoprotein (RNP) scans DNA for a short protospacer-adjacent motif (PAM) directly beside the gRNA binding region of the target site.^73,75–77^ The simplicity of re-targeting the Cas9 protein to these new sites via recoding the spacer of a gRNA enables precise changes to the genome. To overcome limitations in the breadth of editing caused by the requirement for the Cas9 protein to recognize a PAM, new Cas9 and Cas12a proteins with vastly expanded targeting ranges that permit editing of previously inaccessible sequences have been developed.^78–84^

Beyond such traditional nuclease-based editing, cytosine base editors and adenine base editors (ABEs) enable the introduction of precise C-to-T and A-to-G changes, respectively.^78,85–89^ Finally, new CRISPR prime editors (PEs) permit the insertion of custom changes into the genome by using a reverse transcriptase (RT) domain and a PE guide RNA.^90–92^ Collectively, genome editing holds significant breadth and promise as a gene therapy. However, additional considerations are relevant. First, the editing machinery required is relatively large genetically and pushes the cloning capacity of vectors such as AAV. Second, the off-target effects of gene editing technologies need in-depth assessment and quantification as these are lifelong treatment regimens and necessitate the safety assessment to inform risk-to-benefit analysis for the patient.

**Figure 4:**
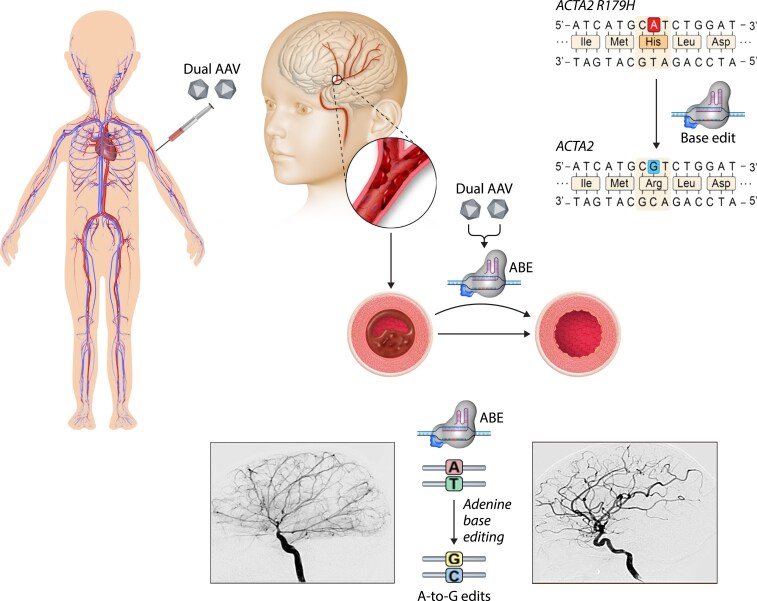
Schematic representation of gene targeted correction using base editing for a severe SMC prototypical vascular disease multisystem smooth muscle dysfunction syndrome (MSMDS). Systemic intravenous delivery of CRISPR/Cas9 Nickase ABE using a dual AAV approach. In this, two AAVs are necessary due to the genome space limitations of AAV being insufficient for some gene editing machinery. This in turn requires both AAVs to be in the same cell in order to correct the most prevalent MSMDS variant of ACTA2 (the arginine 179 to histidine mutation). Following transduction of vascular smooth muscle cells *in vivo* but the AAVs, ABE restores healthy sequence and prevents/ameliorate cerebral arteriopathy.

In the cardiovascular system, broad classes of diseases caused by missense variants are preferred candidates for gene editing. The gene editors are applied to correct or to knock down pathogenic variants where therapeutic effect relies on eliminating the mutant deleterious protein [e.g. ACTA2-related vasculopathy (see *Figure 4*)], CADASIL, COL4A1- and COL4A2-related vascular disorders, Loeys–Dietz syndrome, Myhre syndrome, Marfan’s disease, and hereditary haemorrhagic telangiectasia or correction or exon-skipping strategies to restore loss-of-function variants [i.e. arterial calcification due to deficiency of CD73, generalized arterial calcification of infancy, pseudoxanthoma elasticum, X-linked recessive diseases such as DMD, X-linked Emery–Dreifuss muscular dystrophy (EDMD), Barth syndrome, and familial forms of hypercholesterolaemia]. These approaches must be coupled to existing gene delivery technology, and we provide exemplars for the heart, the liver [largely taking advantage of multi-valent N-acetylgalactosamine (GalNAc) targeting ligand technology], and the vasculature.

In cardiac disease, base editing shows efficiency in experimental models in correcting nonsense pathogenic variants in DMD mice.^93^ Systemic delivery of AAV9-ABE-SpCas9-NG^81^ resulted in a near complete rescue of dystrophin in *mdx^4cv^* mouse hearts.^93^ The off-target activities in both gene editing studies remained low.^94^ Chai *et al*.^95^ used CRISPR/Cas9-ABE to correct a pathogenic variant of hypertrophic cardiomyopathy, the β-myosin (*MYH7*) gene (c.1208 G>A: p.Arg403Gln). Intra-thoracic injection of dual-AAV9 virus resulted in a dramatic reduction of hypertrophy and fibrosis.

CRISPR-Cas base editors have also shown efficiency in ablating the expression of PCSK9 (proprotein convertase subtilisin/kexin type 9) and reducing LDL cholesterol levels in the liver.^39^ The development of LDL receptor independent delivery using the GalNAC lipid nanoparticles technology has enabled both delivery of genome editors to patients with intact LDL receptor activity as well as to those afflicted by homozygous familial hypercholesterolaemia. GalNAC lipid nanoparticles technology also increases the editing efficiency of hepatocytes with minimal editing in non-targeted tissues.^96^ Emerging results from clinical trials of *in vivo* liver editing for hereditary forms of hypercholesterolaemia also highlight the challenges of monitoring *in vivo* editing off-target effects^97^ which is a key factor in risk-to-benefit analysis.

In vasculopathies, most developments are at the experimental stage, for example, recent developments in experimental *in vivo* gene editing targeting the ACTA2 vasculopathy multisystem smooth muscle dysfunction syndrome (MSMDS) using systemically injection of AAV vectors^98^ (see *Figure 4*). Hutchinson–Gilford progeria syndrome has also been targeted experimentally using base editing. Most cases of Hutchinson–Gilford progeria syndrome are caused by the c.1824 C>T *de novo* pathogenic variants in the *LMNA* gene, encoding lamin A/C proteins. As in many diseases caused by dominant-negative missense variants, one approach is to selectively downregulate the mutant protein (allele) which could make the therapy more widely available. However, ameliorating disease progression is highly dependent on understanding several levels of gene regulation, adaptive cellular mechanisms, and the biology or haplosufficiency of the specific protein. As example, in Hutchinson–Gilford progeria syndrome, the incomplete reduction of progerin and the concomitant reduction of lamin A protein showed a limited effect (25% increase in lifespan) and unintended consequences while normalization of DNA sequence by base editing resulted in up to 2.4-fold longer survival and was well tolerated.^99^

How will future approaches enhance the potential of gene editing in the cardiovascular system? Aside from improvement in delivery and control of expression of gene editing machinery, the assessment of off-target mutagenesis, a critical concern in genome editing, has been dramatically improved by the development of new methods for unbiased identification of genome editing activity on and off target.^100–105^ Various measures, including engineered Cas9 variants with enhanced on/off-target specificity, optimized gRNA design, chemical modification of gRNAs, and co-administration of catalytically inactive guide RNAs, have been implemented.^89,106–110^ Potential deleterious effects such as large deletions, inversions at on-target sites, and integration of the AAV viral genome at Cas9-induced double-strand breaks highlight the need for comprehensive safety measures required by the regulatory agencies.

## 5. Regulating expression of therapeutic genes

**Figure 5:**
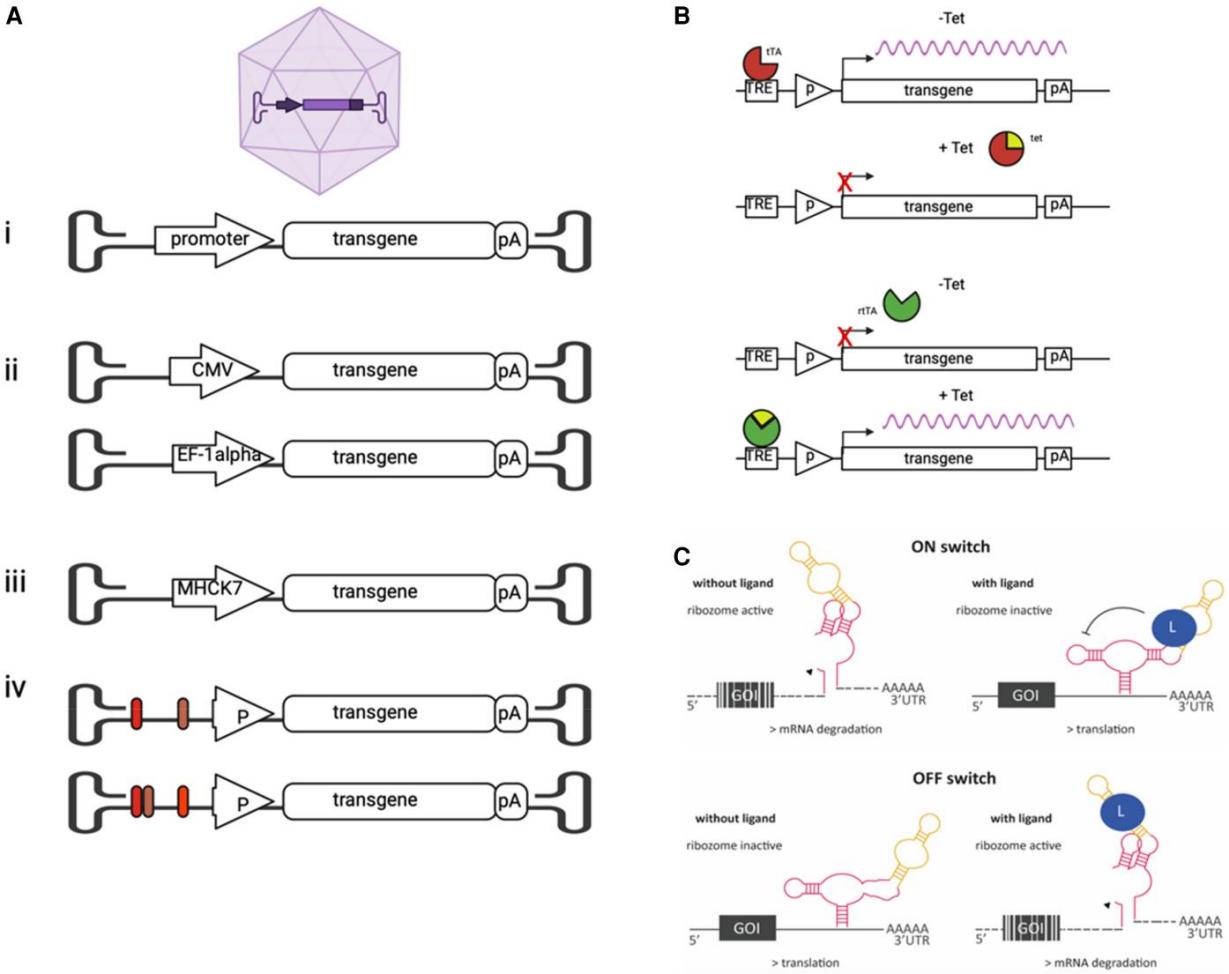
Designs of synthetic promoters for cardiovascular gene therapy (*A*). Example cartoon designs of AAV. (i) Gene therapy promoters represent a typical generic gene therapy cassette design consisting of a promoter, a transgene, and poly A sequence. (ii) Gene therapy cassette with standard constitutive promoters CMV and EF1-alpha. (iii) Gene therapy cassette with a muscle specific promoter MHCK7 enabling genes to be expressed in cardiac muscle. (iv) Synthetics promoters consisting of combinations of cis regulatory elements (coloured bars) such as enhancer sequences upstream of a mini CMV promoter that can be screened for tissue specific regulation of a transgene. (*B*) Cartoon of a tetracycline OFF/ON system. In the tetracycline (tet) OFF system, tTA is a tetracycline controlled activator (green protein) created by fusing the protein tetR with the C-terminal domain of VP16 a transcriptional activation domain from herpes simplex virus. In the absence of tetracycline (yellow triangle), the tetR portion of tTA will bind to the TRE region consisting of a series of tet operator (tetO) sequences and the activation domain promotes expression. In the presence of tetracycline, tetracycline binds to tetR. This prevents tTA binding to the tetO sequences resulting in reduced gene expression. In the tet ON system, tetR was mutated leading to the development of a reverse Tet repressor rTetR (red protein) which relies on the presence of tetracycline (yellow triangle) for induction, rather than repression. (*C*) Cartoon showing aptazyme-based regulation of mRNA stability of a gene of interest (GOI). Ribozymes (illustrated in red) are self-cleaving RNAs and aptamers (illustrated in yellow) are RNAs that bind to specific ligands (illustrated by blue circles). When the two sequences are combined, they result in an aptazyme which is an RNA sequence whose cleavage is regulated by the presence or absence of a ligand. When an aptazyme is engineered into the untranslated region (UTR) of an mRNA depending on the conformation, it can act as either an ON switch or an OFF switch. In the ON conformation, the binding of the ligand inactivates the cleavage of the RNA and thus prevents RNA degradation allowing the protein to be expressed. In the OFF conformation, the binding of the ligand enables the ribozyme activity cleaving the RNA resulting in the degradation of the mRNA transcript decreasing protein expression of the gene of interest (GOI).

A key challenge for the clinical gene therapy is the control of transgene expression (see *Figure 5*). On the one hand, overexpression can be detrimental, but on the other hand, sufficient gene expression is needed to achieve efficacy. Use of promoters based on transcription in viral systems (such as cytomegalovirus) is commonplace and powerful, yet non-specific. Mammalian promoters and cell-specific promoters are also used to help control expression effectively. Each therapeutic indication likely requires a bespoke design to refine transcriptional activities to compartmentalize expression. For example, improving cardiomyocyte calcium handling in heart failure would require long-term and consistent expression of the therapeutic gene. Conversely, expression of a pro-regenerative gene in cardiomyocytes after MI would require precise control, likely short term, and then shut down. Here, we recap on current state-of-the-art and briefly highlight future opportunities that are likely to be incorporated into design of cardiovascular gene medicines.

Promoters are regulatory DNA elements located upstream of a gene coding region. Promoter activity is regulated by transcription factors (TFs) that recognize specific DNA elements at TF binding sites.^111^ ‘Synthetic’ promoters (*Figure 5A*) can be built by bringing together TF binding sites arrayed immediately upstream of a minimal promoter, which is required to initiate transcription of the gene. TF binding activates the core promoter, driving transcription. This approach has been used to produce promoters that function specifically in various cell types, such as hepatocytes, myocytes, and muscle.^112–114^ Identification and screening for synthetic promoters and enhancers involves building libraries of DNA parts (containing the TF binding sites) and screening for the desired activity. Long-range cis-regulatory elements (called ‘enhancers’) can provide the selectivity for a particular cell.^115^ Recent technological advances include mapping of TFs using ChIPseq, enhancer chromatin markers such as H3K27 acetylation,^116^ and chromatin accessibility (ATAC-seq)^117^ enabling the more efficient identification of cell type-specific DNA sequences.

What is being developed that can go beyond state-of-the-art? New approaches have been developed to engineer ‘sense and respond’ circuits that recognize the specific disease state and precipitate a therapeutic response. Relevant cardiometabolic examples include insulin resistance,^118^ obesity,^119^ and hypertension^120^ (reviewed in Xie *et al*.^121^) where the promoter element responds to cues within the body with increased or decreased expression of the therapeutic gene. Other systems have been developed to enable exogenous small molecule control of gene expression which could potentially act as safety switches in control of therapeutic expression levels. A classic example is the so called Tet-ON and Tet-OFF system that is responsive to doxycycline and enables induction or inhibition of transgene expression in response to drug addiction or removal, respectively.^122^ The advantage here is that technology could allow genes to be switched on or off in a repetitive and controlled manner by small molecule administration following gene therapy (*Figure 5B*). This might have particular benefit for gene therapies that instil growth, like pro-regenerative or pro-reparative therapies. Exogenous control of gene therapy expression can also be controlled by light^123,124^ and by acoustics,^125^ but their use in mainstream cardiovascular gene therapy has yet to be consolidated.

RNA-based regulatory devices are also an exciting alternative to protein-based systems (*Figure 5C*). A riboswitch is a regulatory section of mRNA located in either the 5′ or 3′ untranslated (UTR) region that specifically interacts with a trigger molecule resulting in a change in mRNA structure or stability changing the level of production of the protein encoded by the mRNA. Riboswitches responding to various ligands including small molecules such as tetracycline, theophylline, and aminoglycosides have been designed. The use of riboswitch regulation has been reported for a range of mouse tissues, including the heart.^126,127^ These adaptions for transgene control are important components of clinically optimized gene therapy systems.

## 6. Conclusion

**Figure 6:**
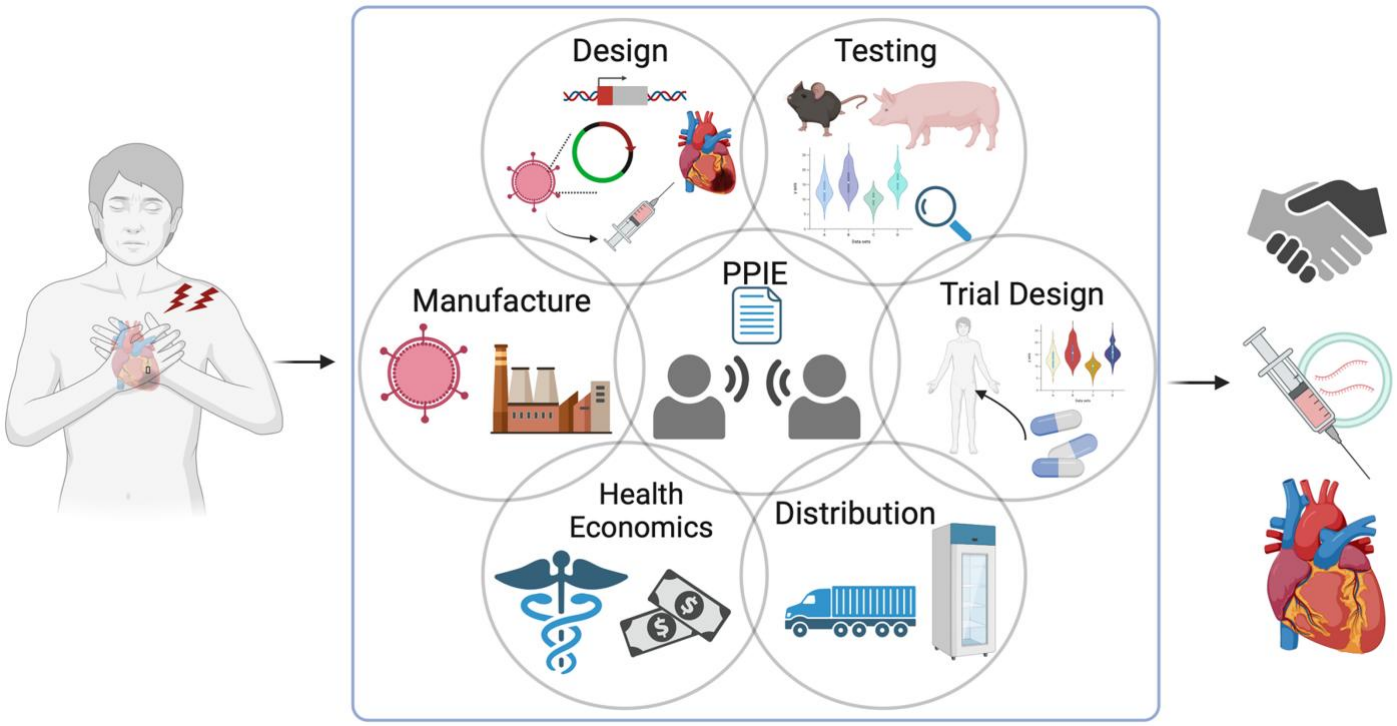
Creating effective research environments for development of gene therapy advanced medicinal products. Acceleration in the pace of development of cardiovascular gene therapy products requires the implementation of interdisciplinary ecosystems. Integrating diverse skill sets can ensure harmony across the preclinical-clinical testing pipeline and onwards towards product licensing and routine clinical care.

The first approved gene therapy medicine in the cardiovascular system is a promising goal. The field remains buoyant with many approaches being tested in clinical trials. Novel technologies are improving local and systemic delivery to the heart and vasculature, improving the potential efficacy and safety of existing approaches. Advances in gene editing are enabling improvements in monogenic cardiovascular disease gene therapy. Refining control of gene transcription is informing the field as to how best optimize therapeutic gene expression. Improvements in the efficiency of dosing, safety, and efficacy and, potentially, lower costs are key priorities for further research and working in defined and focused interdisciplinary ecosystems across stakeholders will accelerate pace of development (*Figure* *6*). In conclusion, there are genuine grounds for optimism that gene therapy will translate into benefits for patients within the decade.

## Data Availability

No new data were generated or analysed in support of this research.
